# Associations of *ASIC2*, *IKZF3*, and *GSDMB* Variants with Gestational Diabetes Mellitus and Their Interactions with Environmental Exposures Among Chinese Women

**DOI:** 10.3390/biom16091373

**Published:** 2026-09-21

**Authors:** Song Wang, Xiao Huang, Xiaoxu Shen, Yi Zhang, Lin Tao, Yuanzhong Zhou, Hongsong Yu, Xin Wang

**Affiliations:** 1 School of Basic Medical Science, Special Key Laboratory of Gene Detection and Therapy of Guizhou Province, Zunyi Medical University, Zunyi 563000, Chinahuangxiao459@163.com (X.H.);; 2 Department of Immunology, Key Laboratory of Cancer Prevention and Treatment of Guizhou Province, Zunyi Medical University, Zunyi 563000, China; 3 School of Public Health, Key Laboratory of Maternal & Child Health and Exposure Science of Guizhou Higher Education Institutes, Zunyi Medical University, Zunyi 563000, China

**Keywords:** GDM, genetic variants, environmental factors, interactions, risk

## Abstract

Gestational diabetes mellitus (GDM) is a common pregnancy complication associated with adverse maternal and offspring cardiometabolic outcomes. However, the combined effects of genetic susceptibility and environmental exposures on GDM risk remain unclear. We conducted a case–control study of 1564 pregnant women in China. Targeted next-generation sequencing was used to genotype *ASIC2*/rs12450465, *IKZF3*/rs2060941, and *GSDMB*/rs2305479. Associations of individual variants and haplotypes with GDM were evaluated, and interactions with exposure to PM_2.5_, O_3_, and monoisobutyl phthalate (MIBP) were assessed. Multiple comparisons in the primary covariate-unadjusted analyses were controlled using the Benjamini–Hochberg false discovery rate (FDR) procedure. In the overall study population, the rs12450465-rs2060941-rs2305479 T-T-T haplotype was significantly associated with GDM after FDR correction (nominal *p* = 0.00266, FDR-adjusted *p* = 0.0133, OR = 1.741, 95% CI = 1.208–2.509). Several gene–gene and gene–air pollutant interaction signals, particularly those involving O_3_ exposure, remained statistically significant after FDR correction in the primary covariate-unadjusted analyses. Among 289 participants with urinary MIBP measurements, the T-T-T haplotype was similarly associated with GDM (nominal *p* = 0.003837, FDR-adjusted *p* = 0.0192, OR = 2.980, 95% CI = 1.378–6.446), and additional interaction signals between this haplotype and MIBP exposure were identified. These findings suggest that gene–environment interactions may contribute to susceptibility to GDM and identify the T-T-T haplotype as a potential susceptibility marker. Nevertheless, the results, particularly those from the smaller MIBP subgroup and the covariate-unadjusted interaction analyses, require validation in larger, independent, and more diverse populations.

## 1. Introduction

Gestational diabetes mellitus (GDM), marked by reduced ability to process glucose, is usually identified in expectant mothers [1,2,3]. Evidence suggests that in early pregnancy, insulin secretion generally increases, while insulin sensitivity may remain stable, decrease, or, in some cases, improve. As pregnancy progresses, particularly during the second trimester, insulin sensitivity typically declines, reaching its lowest point in the third trimester. After delivery, insulin sensitivity usually improves with the detachment of the placenta [4]. Historically, GDM has been associated with complications during pregnancy and for newborns. Recent studies suggest that GDM not only heightens the risk of immediate complications but also potentially impacts long-term cardiovascular and metabolic health outcomes for both mothers and their children [2,5,6,7,8]. Key risk factors for GDM identified in the literature include advanced maternal age, genetic predisposition, exposure to particulate matter (PM), and the use of products containing monoisobutyl phthalate (MIBP) [9,10,11].

Advancements in genetic research have shifted the focus from pregnancy-related risks to identifying key biomarkers linked to diabetes. One potential area of investigation involves acid-sensing ion channels (ASICs), a family of five genes (*ASIC1* to *ASIC5*) that are primarily expressed in the nervous system [12]. Research has demonstrated significant alterations in ASIC expression in the lower thoracic dorsal root ganglion neurons of mice with late-stage type 1 diabetes [13]. Additionally, women carrying certain variants of the *ASIC2* gene exhibit higher genetic risk scores, which are correlated with increased insulin resistance. Notably, a low-energy diet does not mitigate this risk [14].

The *IKZF3* gene, located on chromosome 17q12-q21.1, spans 104 kb and consists of nine exons [15]. It is crucial for the development and differentiation of B lymphocytes and has been associated with an elevated risk of autoimmune diseases, including type 1 diabetes and systemic lupus erythematosus [16,17]. Another important area in diabetes research is the Gasdermin (GSDM) family, which includes six proteins: GSDMA, GSDMB, GSDMC, GSDMD, GSDME/DNFA5, and PVJK/GSDMF [18,19]. These proteins are pivotal in pyroptosis, a form of programmed cell death characterized by cell membrane rupture and inflammation, by regulating membrane permeability. The pore-forming properties of gasdermins have been implicated in various genetic and autoinflammatory conditions [18], as well as in cancer [20,21,22]. Notably, the involvement of the *GSDMB* gene in the pathogenesis of type 1 diabetes was first identified by Saleh et al. [23], and further research by Kaur et al. highlighted its relevance in both type 1 and type 2 diabetes [24]. In contrast, environmental pollutants such as particulate matter (PM) and phthalates (PAEs) have also been linked to an increased risk of GDM [25]. PAEs, commonly used in various industrial products, can disrupt the endocrine system, and prolonged or high-concentration exposure may lead to serious health issues [26,27,28]. PAEs can be divided into two groups according to their molecular weight: low-molecular-weight compounds (e.g., diethyl phthalate, dibutyl phthalate, and diisobutyl phthalate) and high-molecular-weight compounds (e.g., benzyl butyl phthalate, di(2-ethylhexyl) phthalate, and diisononyl phthalate) [29,30]. For example, diisobutyl phthalate (DIBP) undergoes hydrolysis in the liver by esterases to produce two primary metabolites: MIBP and isobutanol. MIBP is further metabolized through processes like oxidation, hydroxylation, or conjugation, resulting in more polar metabolites that are primarily excreted in urine. In biological monitoring, DIBP metabolites, particularly MIBP, are used as key biomarkers for assessing exposure levels. Recent findings have revealed a significant association between MIBP levels and the occurrence of GDM [31,32,33]. However, further research is needed to clarify the precise roles of various PAE metabolites in GDM development and to uncover the associated biological mechanisms.

Accumulating evidence suggests that genetic susceptibility and exposure to environmental chemicals contribute to the development of GDM. However, the extent to which genetic variants interact with environmental exposures to influence GDM risk remains unclear. This study investigated the individual and joint associations of variants in *ASIC2*, *IKZF3*, and *GSDMB* with GDM, with particular attention to their interactions with exposure to PM_2.5_ and O_3_, and MIBP. The findings may improve our understanding of gene–environment interactions in GDM and provide a basis for further validation in independent populations.

## 2. Materials and Methods

### 2.1. Study Participants

Pregnant women enrolled in this study were recruited from Beijing, Wuhan in Hubei Province, and Zunyi in Guizhou Province. GDM status was determined at 24–28 gestational weeks using a 75 g oral glucose tolerance test in accordance with the International Association of Diabetes and Pregnancy Study Groups (IADPSG) recommendations. Participants maintained a regular diet containing no less than 150 g of carbohydrates per day for three days before testing and underwent an overnight fast of 8–10 h. The glucose load, consisting of 75 g of anhydrous glucose dissolved in 300 mL of water, was consumed within 5 min. Venous blood was collected in the fasting state and at 1 and 2 h after glucose administration for determination of plasma glucose concentrations. Participants were classified as having GDM when at least one glucose measurement reached the corresponding diagnostic cut-off (fasting, 5.1 mmol/L; 1 h, 10.0 mmol/L; or 2 h, 8.5 mmol/L); those without abnormal glucose values were assigned to the healthy control group. Individuals with diabetes diagnosed before pregnancy, multiple gestation, gestational complications or endocrine abnormalities influencing glucose homeostasis, major cardiovascular, liver, kidney, or psychiatric diseases, or immune-related disorders were not eligible for inclusion. Initially, 1566 women fulfilled the study requirements [10,34]; however, two participants were excluded because complete genotype information was unavailable for all target SNPs included in the genetic analyses. Consequently, 1564 women were included in the subsequent analyses, comprising 701 participants with GDM and 863 healthy controls. All participants provided written informed consent, and the study protocol was reviewed and approved by the Ethics Committee of Zunyi Medical University. The study was registered with the Chinese Clinical Trial Registry (ChiCTR2000029178).

### 2.2. Assessment of Air Pollution Exposure

Daily maximum 8 h average O_3_ levels and daily average PM_2.5_ levels were obtained from the TAP website (http://tapdata.org.cn/, accessed on 17 August 2026), which provides data at a spatial resolution of 10 km × 10 km. Exposure estimates throughout pregnancy were obtained for each participant, and the mean concentrations over the pregnancy period were used to characterize individual exposure levels. PM_2.5_ and O_3_ exposures were categorized into tertiles based on their respective distributions among the study participants. For PM_2.5_, the low-, medium-, and high- exposure groups were defined as ≤40.77, 40.77–46.25, and >46.25 μg/m^3^, respectively (overall range: 22.34–75.51 μg/m^3^). The corresponding categories for O_3_ were ≤87.88, 87.88–95.96, and >95.96 μg/m^3^, respectively (overall range: 66.98–112.56 μg/m^3^).

### 2.3. Selection of SNPs

A candidate-based approach was used for SNP selection. Candidate genes were identified on the basis of published genetic and functional evidence implicating them in metabolic regulation, insulin resistance, diabetes, or immune processes potentially relevant to GDM. Representative evidence supporting the selection of these genes is summarized in Supplementary Table S1. Information on variants within the candidate genes was obtained from the published literature and publicly available genetic databases, including the National Center for Biotechnology Information (NCBI) databases. On this basis, *ASIC2* rs12450465, *IKZF3* rs2060941, and *GSDMB* rs2305479 were selected for genotyping and subsequent association analyses.

### 2.4. DNA Sample Extraction and Sequencing

Genomic DNA was extracted and purified from blood samples using the Magnetic Universal Genomic DNA Extraction Kit (DP705, Tiangen Biotech Co., Ltd., Beijing, China) and stored in sterile containers at 4 °C. Next-generation sequencing was used for in-depth analysis of the target genes. Specific probes were employed for targeted sequencing to determine genotypes and identify SNPs at each locus. Sequencing data were aligned to a reference genome for variant identification and annotation. All procedures were conducted using the Illumina high-throughput sequencing platform to ensure data accuracy and quality. Sequencing was performed by iGeneTech Bioscience Co., Ltd. (Beijing, China), with a sequencing depth of 100×.

### 2.5. MIBP Concentration Measurement

Based on the availability of urine samples, 289 participants, including 192 healthy controls and 97 women with GDM, were included in the MIBP analysis. MIBP levels were quantified using a gas chromatography–triple quadrupole tandem mass spectrometer (GC–MS/MS, Agilent 7010B, Agilent Technologies, Santa Clara, CA, USA). To account for variations in urine dilution, urinary creatinine concentrations were measured using an AU5800 analyzer (Beckman Coulter, Brea, CA, USA). MIBP concentrations were normalized to urinary creatinine and expressed as μg/g creatinine. Creatinine-normalized MIBP concentrations were calculated as follows: MIBP concentration (μg/L)/[urinary creatinine concentration (mmol/L) × 113.12 (g/mol) × 10^−3^].

### 2.6. Statistical Data Analysis

Statistical analyses were performed using SPSS 29.0 (SPSS, Inc., Chicago, IL, USA). Continuous variables were compared between groups using the independent-samples *t* test or the rank-sum test, as appropriate. Associations between individual SNPs and GDM were evaluated under allelic, dominant, recessive, homozygous, and heterozygous genetic models. Haplotype frequencies were estimated, and their associations with GDM were assessed using SHEsis (http://analysis.bio-x.cn/myAnalysis.php, accessed on 17 August 2026).

Unconditional binary logistic regression was used to evaluate the associations of genotypes, haplotypes, and environmental exposures with GDM. Multiplicative gene–gene and gene–environment interactions were assessed by including the corresponding product terms in the regression models. Effect estimates are reported as odds ratios (ORs) with 95% confidence intervals (CIs).

The primary analyses were conducted without covariate adjustment. As sensitivity analyses, multivariable logistic regression models were additionally adjusted for maternal age, pre-pregnancy body mass index (BMI), and recruitment region; these results are presented in Supplementary Tables S2 and S3. Multiple comparisons in the primary unadjusted analyses were controlled using the Benjamini–Hochberg false discovery rate (FDR) procedure within prespecified analytical families. For the primary analyses, both nominal and FDR-adjusted *p* values are reported. For the multivariable sensitivity analyses, *p* values derived from the covariate-adjusted models are presented. All tests were two-sided. A nominal *p* value < 0.05 was considered nominally significant, whereas an FDR-adjusted *p* value < 0.05 was considered statistically significant after correction for multiple comparisons. Additional details on the statistical procedures and the definition of each analytical family are provided in the Supplementary Methods.

## 3. Results

### 3.1. Demographics and Clinical Characteristics

The analysis included 1564 pregnant women, of whom 701 had GDM and 863 served as controls. Maternal age, height, and gestational age did not differ significantly between the two groups (all *p* > 0.05). In contrast, pre-pregnancy BMI was associated with higher odds of GDM (*p* = 0.00019; OR = 2.565, 95% CI: 1.539–4.275) (Table S4).

### 3.2. Allele and Genotype Distribution of SNPs and Their Correlation with GDM Risk

The genetic association analyses included 687 participants recruited in Zunyi (323 GDM cases and 364 controls), 641 in Wuhan (235 GDM cases and 406 controls), and 236 in Beijing (143 GDM cases and 93 controls). The genotype distributions of *ASIC2* rs12450465, *IKZF3* rs2060941, and *GSDMB* rs2305479 are shown in Figure S1. In the control group, the genotype distributions of all three SNPs were consistent with Hardy–Weinberg equilibrium (HWE) (Table S5). Given the multicenter recruitment, genotype and allele frequencies stratified by recruitment city and GDM status are further presented in Supplementary Table S6.

In the unadjusted primary analyses, five genetic models were applied: dominant, recessive, allelic, heterozygous, and homozygous (Table S7). The recessive model for rs12450465 showed an association with GDM risk: pregnant women with the TT genotype had a higher estimated risk of GDM compared with those with the CT or CC genotypes (OR = 1.262, 95% CI = 1.029–1.548; nominal *p* = 0.026). For rs2060941, women with the GT or TT genotypes showed a higher estimated risk of GDM than those with the GG genotype under the dominant model (OR = 1.361, 95% CI = 1.007–1.839; nominal *p* = 0.044), and a similar pattern was observed under the heterozygous model (OR = 1.380, 95% CI = 1.015–1.877; nominal *p* = 0.039). For rs2305479, higher estimated GDM risk was observed under both the dominant model (OR = 1.229, 95% CI = 1.006–1.501; nominal *p* = 0.043) and the heterozygous model (OR = 1.243, 95% CI = 1.009–1.531; nominal *p* = 0.041). These nominal associations were attenuated after Benjamini–Hochberg FDR correction across the 15 gene–genetic model tests and did not meet the FDR-adjusted significance threshold (Table S7).

### 3.3. Interaction of SNPs and GDM Risk

**Figure 1:**
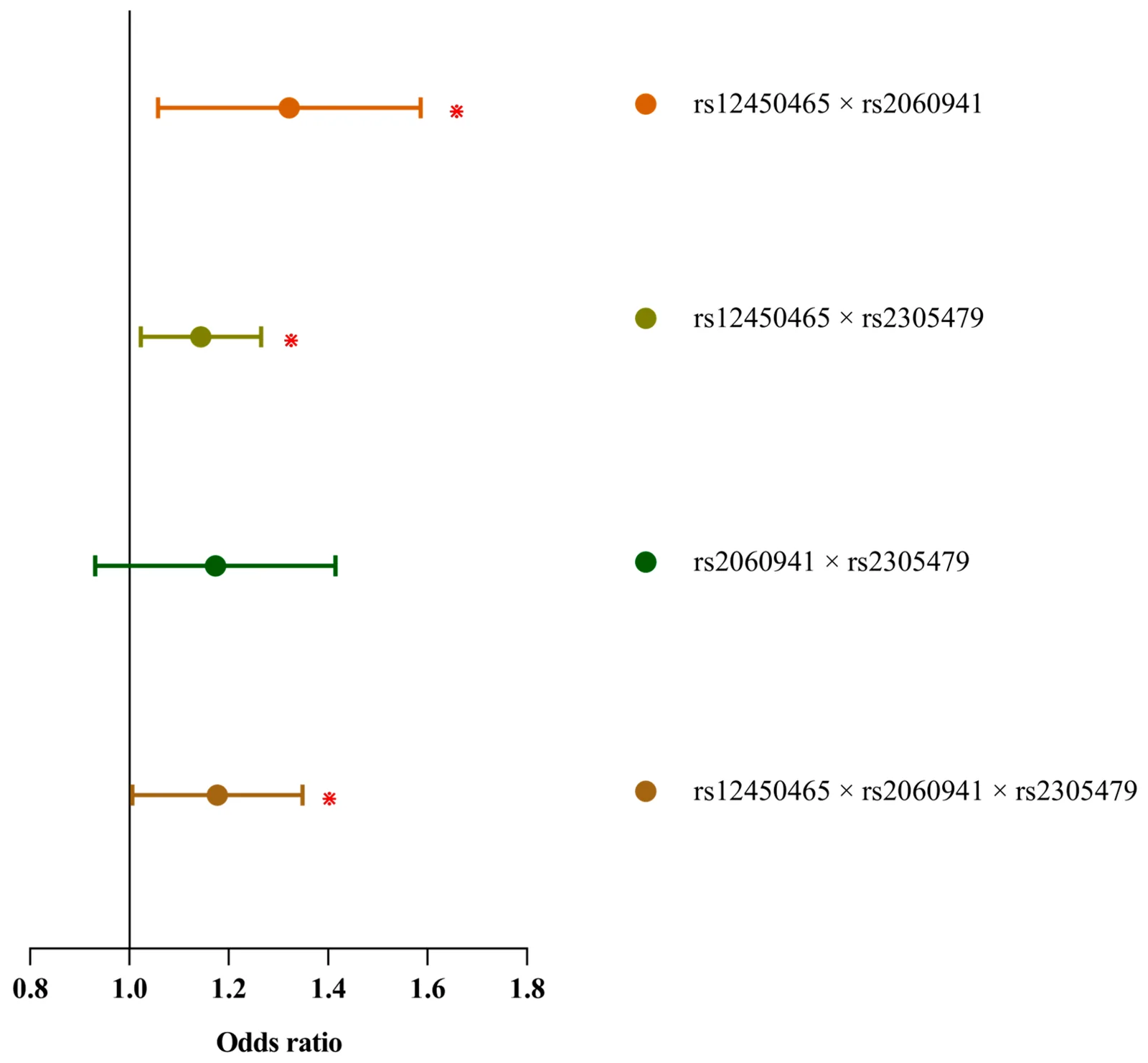
Associations of gene–gene interaction terms with GDM. Odds ratios and 95% confidence intervals were estimated using binary logistic regression in the unadjusted primary analyses. Asterisks indicate interaction terms that remained statistically significant after Benjamini–Hochberg FDR correction (FDR-adjusted *p* < 0.05).

Significant multiplicative interaction terms were observed between rs12450465 and rs2060941, as well as between rs12450465 and rs2305479, in the unadjusted primary analyses (Table 1 and Figure 1). Additionally, the multiplicative interaction term involving all three SNPs (rs12450465, rs2060941, and rs2305479) was also statistically significant (rs12450465 × rs2060941: nominal *p* = 0.010, FDR-adjusted *p* = 0.032, OR = 1.304, 95% CI = 1.066–1.594; rs12450465 × rs2305479: nominal *p* = 0.016, FDR-adjusted *p* = 0.032, OR = 1.139, 95% CI = 1.025–1.267; rs12450465 × rs2060941 × rs2305479: nominal *p* = 0.037, FDR-adjusted *p* = 0.0493, OR = 1.168, 95% CI = 1.010–1.350). Thus, these three gene–gene interaction terms remained statistically significant after FDR correction. However, no significant multiplicative interaction was identified between rs2060941 and rs2305479 (nominal *p* = 0.170, FDR-adjusted *p* = 0.170). In the multivariable-adjusted sensitivity analyses, the rs12450465 × rs2060941 interaction retained nominal statistical significance, whereas the other gene–gene interaction terms did not retain nominal significance (Table S2).

### 3.4. Associations of Gene–Environmental Exposure Interactions with GDM Risk

We performed a comprehensive risk association analysis examining the interactions between environmental factors and genetic variants for GDM. In the unadjusted primary analyses, significant multiplicative interaction terms were observed between three specific genes and O_3_ exposure (Table S8 and Figure S2). Notably, the multiplicative interaction term between rs12450465 and O_3_ remained statistically significant after FDR correction, whereas the interaction between rs12450465 and PM_2.5_ did not remain statistically significant after FDR correction (rs12450465 × O_3_: nominal *p* = 2.0203 × 10^−7^, FDR-adjusted *p* = 1.82 × 10^−6^, OR = 1.157, 95% CI = 1.095–1.222; rs12450465 × PM2.5: nominal *p* = 0.036, FDR-adjusted *p* = 0.054, OR = 1.061, 95% CI = 1.004–1.121). Similarly, the multiplicative interaction terms between rs2060941 and O3 (nominal *p* = 0.016, FDR-adjusted *p* = 0.0288, OR = 1.170, 95% CI = 1.030–1.328) and between rs2305479 and O3 (nominal *p* = 1.82 × 10^−4^, FDR-adjusted *p* = 0.000819, OR = 1.146, 95% CI = 1.067–1.231) also remained statistically significant after FDR correction. Furthermore, the higher-order interaction term between rs12450465, PM_2.5_, and O_3_ remained statistically significant after FDR correction (rs12450465 × PM_2.5_ × O_3_: nominal *p* = 0.001, FDR-adjusted *p* = 0.003, OR = 1.035, 95% CI = 1.014–1.057). Likewise, the higher-order interaction term between rs2305479, PM_2.5_, and O_3_ remained statistically significant after FDR correction (rs2305479 × PM_2.5_ × O_3_: nominal *p* = 0.012, FDR-adjusted *p* = 0.027, OR = 1.039, 95% CI = 1.008–1.071). However, the interaction between rs2060941, PM_2.5_, and O_3_ was not statistically significant after FDR correction (FDR-adjusted *p* = 0.090). Overall, five gene–air pollutant interaction terms remained statistically significant after FDR correction in the primary analyses. In the multivariable-adjusted sensitivity analyses, the gene–air pollutant interaction terms were not nominally significant (Table S2).

### 3.5. Effect of Haplotypes and Their Interaction with Environmental Exposures (PM_2.5_, O_3_) on GDM Risk

**Figure 2:**
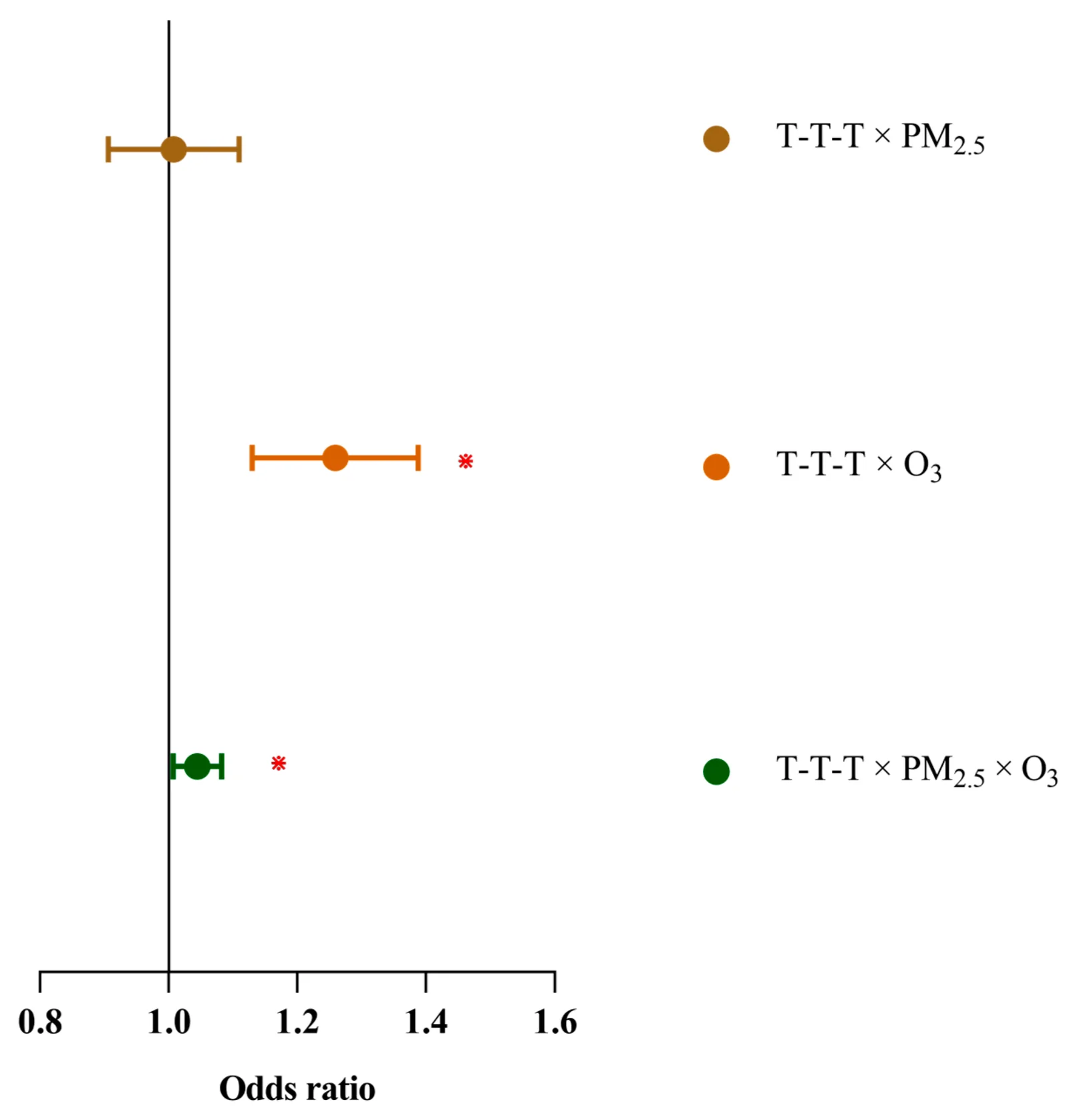
Associations of T-T-T haplotype–environment interaction terms with GDM. Odds ratios and 95% confidence intervals represent regression estimates in the unadjusted primary analyses for interaction terms involving the rs12450465-rs2060941-rs2305479 T-T-T haplotype and PM_2.5_ and/or O_3_. Asterisks indicate interaction terms that remained statistically significant after Benjamini–Hochberg FDR correction (FDR-adjusted *p* < 0.05).

Using the SHEsis program, we analyzed five haplotypes (Table S9 and Figure S3). In the unadjusted primary analyses, the T-T-T haplotype of rs12450465-rs2060941-rs2305479 was significantly associated with GDM after FDR correction (nominal *p* = 0.00266, FDR-adjusted *p* = 0.0133, OR = 1.741, 95% CI = 1.208–2.509), while the other four haplotypes did not show such an association. Further analysis revealed that significant multiplicative interaction terms were observed between the T-T-T haplotype and O_3_ exposure, as well as among the T-T-T haplotype, PM_2.5_, and O_3_ (see Table 2 and Figure 2) (T-T-T × O_3_: nominal *p* = 0.000014, FDR-adjusted *p* = 0.000042, OR = 1.254, 95% CI = 1.132–1.390; T-T-T × PM_2.5_ × O_3_: nominal *p* = 0.019, FDR-adjusted *p* = 0.0285, OR = 1.044, 95% CI = 1.007–1.082). In contrast, the T-T-T × PM_2.5_ interaction alone was not significant (nominal *p* = 0.937, FDR-adjusted *p* = 0.937, OR = 1.004, 95% CI = 0.908–1.111). Both the T-T-T × O_3_ and T-T-T × PM_2.5_ × O_3_ interaction terms remained statistically significant after FDR correction in the primary analyses. In the multivariable-adjusted sensitivity analyses, the T-T-T haplotype–air pollutant interaction terms were not nominally significant (Table S2).

### 3.6. Gene-Internal Exposure (MIBP)-External Exposure (PM_2.5_, O_3_) Interactions and GDM Risk

In the unadjusted primary analyses, MIBP exposure was associated with an OR of 1.002 (95% CI = 1.000–1.004; nominal *p* = 0.034, FDR-adjusted *p* = 0.0884). For rs12450465, the multiplicative interaction estimates were as follows: rs12450465 × MIBP (OR = 1.002, 95% CI = 1.000–1.003; nominal *p* = 0.031, FDR-adjusted *p* = 0.0884), rs12450465 × MIBP × PM_2.5_ (OR = 1.001, 95% CI = 1.000–1.002; nominal *p* = 0.008, FDR-adjusted *p* = 0.0520), rs12450465 × MIBP × O_3_ (OR = 1.001, 95% CI = 1.000–1.002; nominal *p* = 0.012, FDR-adjusted *p* = 0.0520), and rs12450465 × MIBP × PM_2.5_ × O_3_ (OR = 1.001, 95% CI = 1.000–1.001; nominal *p* = 0.008, FDR-adjusted *p* = 0.0520) (Table 3). Although nominal associations were observed for MIBP and several rs12450465–MIBP-related interaction terms, none met the FDR-adjusted significance threshold. In the multivariable-adjusted sensitivity analyses, MIBP remained nominally significant, whereas the gene–MIBP-related interaction terms were not nominally significant (Table S3). Given the limited sample size of the MIBP subset, the higher-order interaction findings should be interpreted as exploratory.

### 3.7. SNP Haplotype Interaction with Internal (MIBP) and External (PM_2.5_, O_3_) Exposure Factors on GDM Risk

**Figure 3:**
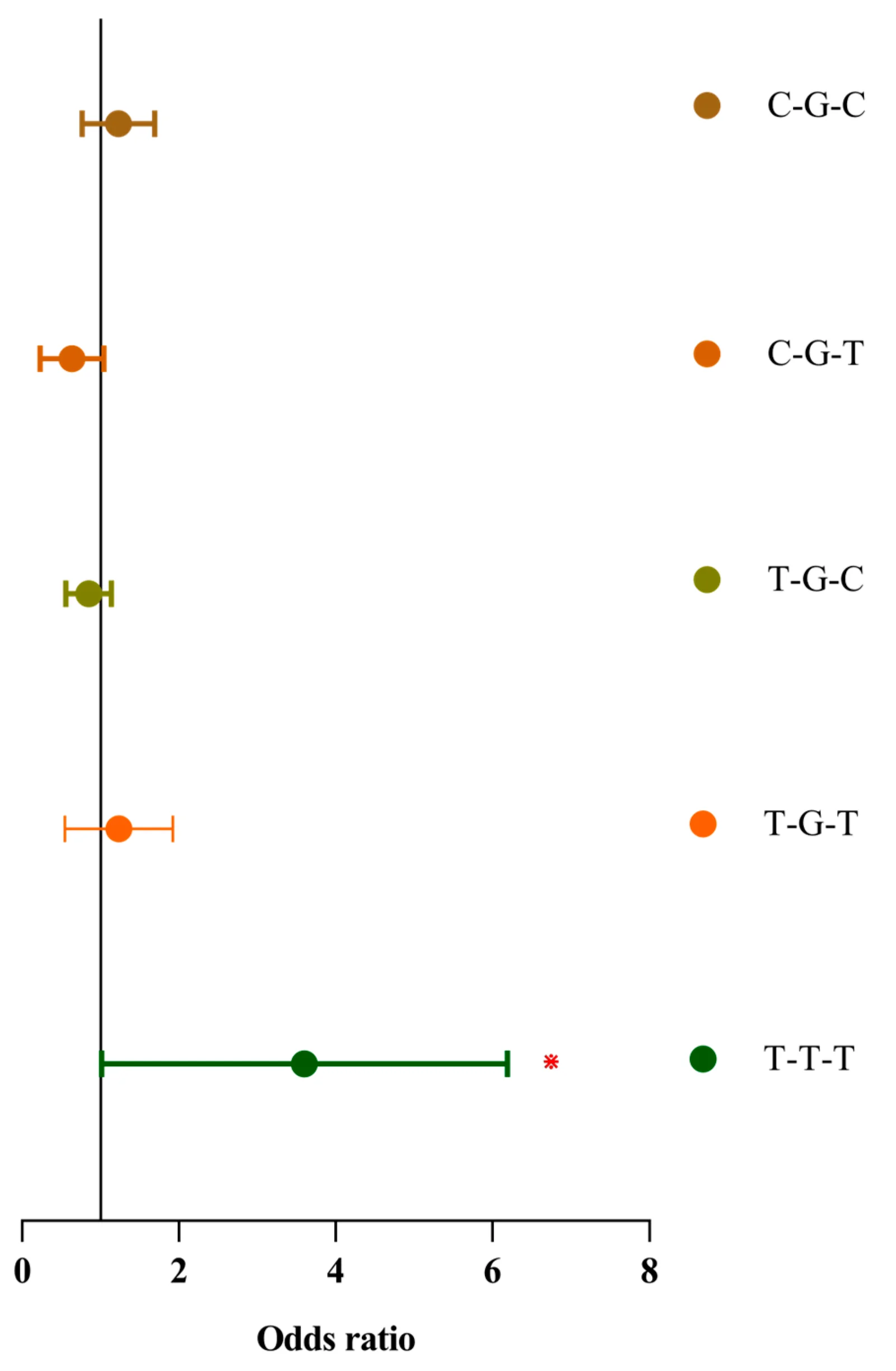
Associations between rs12450465–rs2060941–rs2305479 haplotypes and GDM. Odds ratios and 95% confidence intervals were estimated from the unadjusted primary haplotype association analyses. Asterisks indicate haplotype associations that remained statistically significant after Benjamini–Hochberg FDR correction (FDR-adjusted *p* < 0.05).

**Figure 4:**
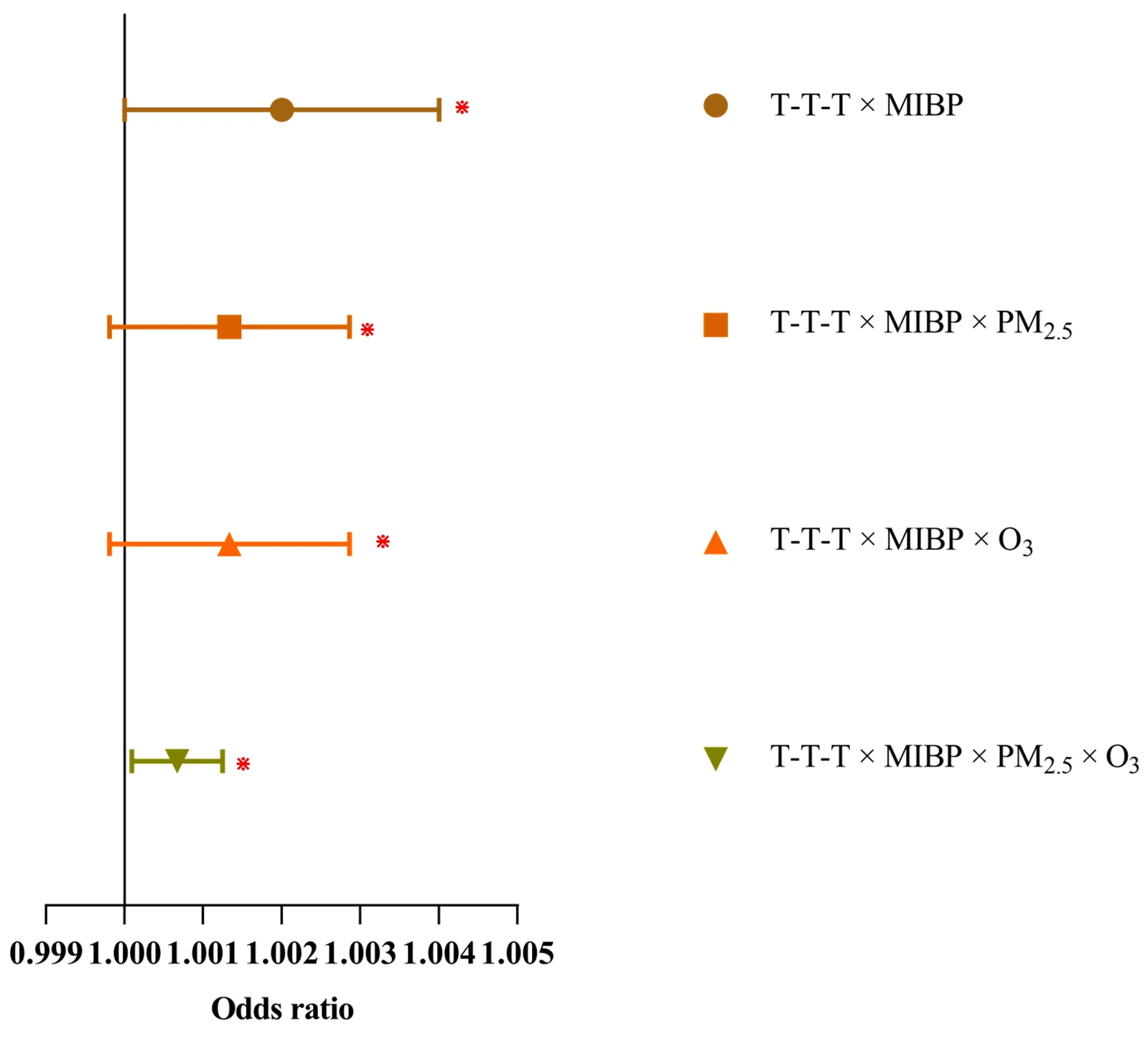
Associations of T-T-T haplotype–MIBP–environment interaction terms with GDM. Odds ratios and 95% confidence intervals represent logistic regression estimates from the unadjusted primary analyses for interaction terms involving the rs12450465–rs2060941–rs2305479 T-T-T haplotype, urinary MIBP, PM_2.5_, and O_3_. Asterisks indicate interaction terms that remained statistically significant after Benjamini–Hochberg FDR correction (FDR-adjusted *p* < 0.05).

Haplotype analysis of 289 participants with MIBP measurement (Table 4 and Figure 3) revealed that the rs12450465-rs2060941-rs2305479 (T-T-T) haplotype was significantly associated with GDM after FDR correction (nominal *p* = 0.003837, FDR-adjusted *p* = 0.0192, OR = 2.980, 95% CI = 1.378–6.446). Interaction analysis (see Table 5 and Figure 4) identified significant multiplicative interaction terms between this haplotype and environmental factors, particularly MIBP, MIBP × PM_2.5_, MIBP × O_3_, and MIBP × PM_2.5_ × O_3_. All four interaction terms remained statistically significant after FDR correction in the unadjusted primary analyses (T-T-T × MIBP: nominal *p* = 0.045, FDR-adjusted *p* = 0.045, OR = 1.002, 95% CI = 1.000–1.004; T-T-T × MIBP × PM_2.5_: nominal *p* = 0.021, FDR-adjusted *p* = 0.0293, OR = 1.001, 95% CI = 1.000–1.003; T-T-T × MIBP × O_3_: nominal *p* = 0.022, FDR-adjusted *p* = 0.0293, OR = 1.001, 95% CI = 1.000–1.003; T-T-T × MIBP × PM_2.5_ × O_3_: nominal *p* = 0.015, FDR-adjusted *p* = 0.0293, OR = 1.001, 95% CI = 1.000–1.001). In the multivariable-adjusted sensitivity analyses, the corresponding T-T-T haplotype–MIBP interaction terms were not nominally significant (Table S3).

## 4. Discussion

This study examined the individual and joint associations of genetic variation and environmental exposures with GDM. The T-T-T haplotype comprising *ASIC2* rs12450465, *IKZF3* rs2060941, and *GSDMB* rs2305479 showed a significant association with GDM that remained significant after FDR correction. Several multiplicative gene–environment interaction terms, particularly those involving O_3_, also remained significant after FDR correction in the primary unadjusted analyses. These findings suggest that joint analyses of genetic variants and environmental exposures may provide information on GDM susceptibility that is not captured by single-variant analyses alone. However, because several interaction signals were attenuated after covariate adjustment, the findings should be interpreted as exploratory.

Recent research by Lizarraga et al. summarized GDM-associated SNPs, epigenetic alterations, and microbiota profiles, suggesting that genetic, epigenetic, and microbial factors may jointly contribute to GDM susceptibility and may also influence offspring health [35]. In addition, a recent study in Chinese women reported that *CDKN2A*/*2B* variants, haplotypes, O_3_ and PM_2.5_ exposure, and their gene–environment interactions were associated with increased GDM risk [36]. Together, these findings support the need to further investigate gene–environment interactions in GDM.

Accumulating evidence suggests that genetic polymorphisms play an important role in susceptibility to GDM [36,37,38,39]. Although variants in *ASIC2*, *IKZF3*, and *GSDMB* have previously been associated with type 1 and type 2 diabetes, their involvement in GDM has not been well characterized. In the present study, variants in all three genes showed nominal associations with GDM under selected genetic models. None of these single-variant associations remained statistically significant after FDR correction, suggesting that their individual effects may be small or that the nominal findings may reflect multiple testing. Larger studies are needed to obtain more precise estimates of these associations. Previous epidemiological studies have associated exposure to PM_2.5_ and O_3_ with GDM risk [10,40,41,42], providing a rationale for examining whether genetic background modifies associations between air pollution and GDM. In the primary unadjusted analyses, several multiplicative interaction terms involving O_3_, including higher-order interactions with rs12450465 and rs2305479, remained statistically significant after FDR correction, whereas the rs12450465 × PM_2.5_ interaction did not remain significant after FDR correction. This pattern raises the possibility that O_3_ exposure may modify associations between the selected genetic variants and GDM. However, the gene–air pollutant and haplotype–air pollutant interaction signals were attenuated in the multivariable sensitivity analyses adjusted for maternal age, pre-pregnancy BMI, and recruitment region. The observed interactions may therefore be sensitive to maternal or regional characteristics and should not be interpreted as definitive evidence of effect modification. While this study focused on specific air pollutants, existing literature suggests that other pollutants, such as sulfate (SO_4_^2−^), nitrate (NO_3_^−^), ammonium (NH_4_^+^), black carbon (BC), organic matter (OM), mineral dust (DUST), and sea salt (SS), are also linked to the incidence of GDM [40]. The precise effects of interactions between these pollutants and genetic factors remain unclear. Future longitudinal studies should investigate gene–environment interactions related to GDM risk, considering these additional pollutants and adequately controlling for potential confounders.

In our analysis of 1564 samples, we identified a significant association between the T-T-T haplotype and GDM in the unadjusted primary analysis after FDR correction. Furthermore, our findings suggest that this haplotype may be involved in multiplicative interactions with environmental factors related to GDM susceptibility. While these results are preliminary and require further validation, future molecular studies could provide valuable insights into the biological mechanisms underlying this association. Exogenous chemicals are widespread in daily life, and current research shows that many of these substances contribute to disease onset by disrupting endocrine functions through enzyme- and receptor-mediated pathways, as well as through epigenetic effects. These disruptions can negatively impact various human body systems, including the reproductive, metabolic, nervous, and immune systems [43,44]. Phthalates were identified as endocrine disruptors in 2016 [45]. Since then, epidemiological studies have linked elevated levels of phthalate metabolites to insulin resistance and GDM [31,46]. Building on this work, our study assessed the interaction between MIBP metabolites and genetic factors influencing GDM risk. In a study involving 192 healthy controls and 97 GDM cases, we also found a significant association between the T-T-T haplotype and GDM after FDR correction, consistent with that observed in the overall study population. Notably, four multiplicative interaction terms involving the T-T-T haplotype, MIBP, and PM_2.5_ and/or O_3_ remained statistically significant after FDR correction in the unadjusted primary analyses. These findings provide a rationale for further investigation of the interplay among phthalate exposure, air pollution, genetic susceptibility, and GDM. Nevertheless, the MIBP analysis was based on a relatively small subset, and the higher-order interaction models may have included limited numbers of participants in some exposure–genotype combinations. The estimates may therefore be unstable, and the results should be considered hypothesis-generating. Larger studies with repeated biomarker measurements and independent replication are required to assess their robustness.

This study has several significant strengths. Primarily, it offers a comprehensive examination of the relationship between variants in the *ASIC2*, *IKZF3*, and *GSDMB* genes and GDM susceptibility, together with gene–gene interactions and haplotype associations. Additionally, it investigates how these genetic variants interact with environmental factors, addressing a critical gap in understanding the interplay between genetic susceptibility and environmental exposures in GDM. The study also evaluated external air pollutants and an internal phthalate biomarker, allowing genetic and environmental factors to be examined jointly. Furthermore, the Benjamini–Hochberg FDR procedure was applied within predefined hypothesis families, and both nominal and FDR-adjusted *p*-values were reported for the primary analyses.

Several limitations should be considered. First, participants were recruited from three geographically distinct Chinese cities (Beijing, Wuhan, and Zunyi) and information on genetic ancestry was unavailable. Although recruitment region was included in the multivariable models, residual population stratification cannot be excluded. This concern is particularly relevant because both genetic background and environmental exposure distributions may differ across regions. Second, the primary analyses were not adjusted for covariates. Several interaction signals did not retain nominal statistical significance in the models adjusted for maternal age, pre-pregnancy BMI, and recruitment region, indicating that the primary results may be sensitive to confounding or regional heterogeneity. Other potentially relevant factors, including socioeconomic characteristics, diet, physical activity, smoking exposure, parity, family history of diabetes, and season of conception, were not comprehensively available for adjustment. Residual confounding therefore remains possible. Third, urinary MIBP was measured in only 289 participants. Selection into this subset may have introduced bias, and the limited sample size reduced the precision of the haplotype–environment and higher-order interaction estimates. A single urinary measurement may also provide an imperfect estimate of phthalate exposure throughout pregnancy because phthalates have relatively short biological half-lives and urinary concentrations can vary over time. Although normalization to urinary creatinine accounted for urine dilution, repeated measurements across pregnancy would provide a more reliable assessment of exposure. Fourth, ambient PM_2.5_ and O_3_ exposure estimates may not fully represent individual exposure. Spatial resolution, residential mobility, time-activity patterns, occupational exposure, indoor pollution, and personal protective behaviors may have introduced exposure misclassification. Categorizing exposure into tertiles may also have reduced information and obscured nonlinear exposure–response relationships. Fifth, although FDR correction was used to address multiple comparisons, the study evaluated several genetic models, haplotypes, pollutants, and higher-order interaction terms. The possibility of false-positive findings remains, particularly for complex interaction models. Conversely, limited statistical power may have reduced the ability to detect modest main effects or interactions. The absence of an independent replication cohort further limits the strength of the conclusions. Finally, the study was based on a candidate-gene design and did not experimentally examine the functions of the selected variants or the mechanisms underlying the observed associations. The findings therefore cannot establish causality or identify the molecular pathways through which the T-T-T haplotype and environmental exposures might influence GDM.

Taken together, the results suggest that the T-T-T haplotype and selected gene–gene and gene–environment interaction terms may be associated with GDM. The persistence of some associations after FDR correction in the primary unadjusted analyses supports further investigation, whereas their attenuation after covariate adjustment underscores the need for caution. Future studies should include larger and more diverse populations, ancestry-informative genetic data, repeated exposure measurements, broader assessment of pollutant mixtures, and more comprehensive control of confounding. Functional and mechanistic experiments will also be required to determine whether the observed statistical interactions reflect biologically meaningful pathways involved in GDM.

## 5. Conclusions

The findings suggest that genetic susceptibility and environmental exposures may jointly contribute to GDM. The T-T-T haplotype comprising *ASIC2* rs12450465, *IKZF3* rs2060941, and *GSDMB* rs2305479, together with several gene–gene and gene–environment interaction terms, remained statistically significant after FDR correction in the primary unadjusted analyses. However, some interaction signals were attenuated after adjustment for maternal age, pre-pregnancy BMI, and recruitment region. These results should therefore be considered preliminary. The T-T-T haplotype is a candidate susceptibility marker that requires validation in larger, independent populations before its clinical relevance can be determined.

## Figures and Tables

**Table 1:** Association between gene–gene interactions and the GDM risk.

Model	Unadjusted OR (95%CI)	Nominal *p*	FDR-Adjusted *p* ^a^
rs12450465 × rs2060941	1.304 (1.066–1.594)	0.01	0.032
rs12450465 × rs2305479	1.139 (1.025–1.267)	0.016	0.032
rs2060941 × rs2305479	1.156 (0.940–1.422)	0.17	0.170
rs12450465 × rs2060941 × rs2305479	1.168 (1.010–1.350)	0.037	0.0493

^a^ FDR-adjusted *p* values were obtained by applying the Benjamini–Hochberg procedure to the nominal *p* values from the unadjusted primary gene–gene interaction analyses.

**Table 2:** Impact of the interaction between the T-T-T haplotype and environmental factors (PM_2.5_, O_3_) on GDM incidence.

Model	Unadjusted OR (95%CI)	Nominal *p*	FDR-Adjusted *p* ^a^
T-T-T × PM_2.5_	1.004 (0.908–1.111)	0.937	0.937
T-T-T × O_3_	1.254 (1.132–1.390)	0.000014	0.000042
T-T-T × PM_2.5_ × O_3_	1.044 (1.007–1.082)	0.019	0.0285

^a^ FDR-adjusted *p* values were calculated from the nominal *p* values of the unadjusted T-T-T haplotype–air pollutant interaction analyses using the Benjamini–Hochberg procedure within this predefined hypothesis family.

**Table 3:** The effect of interactions between rs12450465, rs2060941, rs2305479, and environmental factors (PM_2.5_, O_3_, MIBP) on GDM incidence.

Model	Unadjusted OR (95%CI)	Nominal *p*	FDR-Adjusted *p* ^a^
MIBP	1.002 (1.000–1.004)	0.034	0.0884
rs12450465 × MIBP	1.002 (1.000–1.003)	0.031	0.0884
rs2060941 × MIBP	1.011 (0.996–1.027)	0.142	0.3077
rs2305479 × MIBP	0.999 (0.992–1.005)	0.683	0.7399
rs12450465 × MIBP × PM_2.5_	1.001 (1.000–1.002)	0.008	0.0520
rs2060941 × MIBP × PM_2.5_	1.003 (0.997–1.009)	0.291	0.5395
rs2305479 × MIBP × PM_2.5_	0.999 (0.996–1.002)	0.592	0.7399
rs12450465 × MIBP × O_3_	1.001 (1.000–1.002)	0.012	0.0520
rs2060941 × MIBP × O_3_	1.003 (0.997–1.010)	0.332	0.5395
rs2305479 × MIBP × O_3_	1.000 (0.996–1.004)	0.991	0.9910
rs12450465 × MIBP × PM_2.5_ × O_3_	1.001 (1.000–1.001)	0.008	0.0520
rs2060941 × MIBP × PM_2.5_ × O_3_	1.001 (0.998–1.003)	0.486	0.7020
rs2305479 × MIBP × PM_2.5_ × O_3_	1.000 (0.998–1.002)	0.648	0.7399

^a^ FDR-adjusted *p* values were derived from the nominal *p* values of the unadjusted analyses using the Benjamini–Hochberg procedure.

**Table 4:** Results of the analysis on the association between haplotypes (rs12450465-rs2060941-rs2305479) and the incidence of GDM.

Model	Unadjusted OR (95%CI)	Nominal *p*	FDR-Adjusted *p* ^a^
C-G-C	1.169 (0.797–1.715)	0.424356	0.5304
C-G-T	0.550 (0.280–1.077)	0.077712	0.1943
T-G-C	0.814 (0.574–1.155)	0.248696	0.4145
T-G-T	1.103 (0.616–1.977)	0.740681	0.7407
T-T-T	2.980 (1.378–6.446)	0.003837	0.0192

^a^ FDR-adjusted *p* values were calculated from the nominal *p* values of the unadjusted primary haplotype association analyses using the Benjamini–Hochberg procedure within this predefined hypothesis family.

**Table 5:** The effect of interactions between the T-T-T haplotype and environmental factors (PM_2.5_, O_3_, MIBP) on GDM incidence.

Model	Unadjusted OR (95%CI)	Nominal *p*	FDR-Adjusted *p* ^a^
T-T-T × MIBP	1.002 (1.000–1.004)	0.045	0.045
T-T-T × MIBP × PM_2.5_	1.001 (1.000–1.003)	0.021	0.0293
T-T-T × MIBP × O_3_	1.001 (1.000–1.003)	0.022	0.0293
T-T-T × MIBP × PM_2.5_ × O_3_	1.001 (1.000–1.001)	0.015	0.0293

^a^ FDR-adjusted *p* values were calculated from the nominal *p* values of the unadjusted primary T-T-T haplotype–MIBP–environment interaction analyses using the Benjamini–Hochberg procedure within this predefined hypothesis family.

## Data Availability

The datasets used and/or analyzed during the current study are available from the corresponding author on reasonable request.

## Supplementary Materials

The following supporting information can be downloaded at: https://www.mdpi.com/article/10.3390/biom16091373/s1, Supplementary Methods: Detailed Statistical Analysis; Table S1: Literature-based evidence supporting the selection of candidate genes; Table S2: Sensitivity analyses of gene–gene, gene–air pollutant, and haplotype–air pollutant interactions with GDM in the overall study population (N = 1564); Table S3: Sensitivity analyses of MIBP-related associations and interaction terms with GDM in the MIBP subset (N = 289); Table S4: The influence of physical characteristics on the risk of developing GDM; Table S5: Hardy–Weinberg equilibrium analysis of the three SNPs in the control group; Table S6: Genotype and allele frequencies by recruitment city and GDM status; Table S7: Association of different genetic models of rs12450465, rs2060941, and rs2305479 with GDM risk; Table S8: Results on the impact of gene–environment interactions on the risk of developing GDM; Table S9: Association between rs12450465-rs2060941-rs2305479 haplotypes and GDM incidence; Figure S1: Genotype distribution of rs12450465, rs2060941, and rs2305479 between the GDM and control groups; Figure S2: Associations of gene–environment interaction terms with GDM; Figure S3: Associations of rs12450465-rs2060941-rs2305479 haplotypes with GDM.

## Abbreviations

GDM	gestational diabetes mellitus
SNP	single nucleotide polymorphism
O_3_	ozone
MIBP	monoisobutyl phthalate
PAEs	phthalates
PM	particulate matter
OR	odds ratio
95% CI	95% confidence interval

## References

[1] Saravanan P., Diabetes in Pregnancy Working Group, Maternal Medicine Clinical Study Group, Royal College of Obstetricians and Gynaecologists (2020). Gestational diabetes: Opportunities for improving maternal and child health. Lancet Diabetes Endocrinol..

[2] Sweeting A., Wong J., Murphy H.R., Ross G.P. (2022). A Clinical Update on Gestational Diabetes Mellitus. Endocr. Rev..

[3] Skibińska M., Kacerovsky M., Grzesiak M., Wujcicka W.I. (2025). Autoimmune pathogenesis of gestational diabetes mellitus: The risk of progression to type 1 diabetes mellitus. Front. Endocrinol..

[4] Hasain Z., Mokhtar N.M., Kamaruddin N.A., Mohamed Ismail N.A., Razalli N.H., Gnanou J.V., Raja Ali R.A. (2020). Gut Microbiota and Gestational Diabetes Mellitus: A Review of Host-Gut Microbiota Interactions and Their Therapeutic Potential. Front. Cell. Infect. Microbiol..

[5] Yin M., Zhang Y., Li X., Liu S., Huang J., Yu H., Li X. (2024). Adverse effects of gestational diabetes mellitus on fetal monocytes revealed by single-cell RNA sequencing. iScience.

[6] Zhang Y., Angley M., Lu L., Smith B.J., Grobman W., Wylie B.J., Zork N.M., D’Alton M.E., McNeil B., Mercer B.M. (2025). Radon Exposure and Gestational Diabetes. JAMA Netw. Open.

[7] Yuan P., Huang J., Wan J., Yu L., Li J., Huang B., Li N., Wei H., Kong L., Qin J. (2026). Trimester-specific gestational weight gain and adverse outcomes in GDM women: A retrospective cohort study. Front. Endocrinol..

[8] Hepp P., Unverdorben L., Hutter S., Kuhn C., Ditsch N., Groß E., Mahner S., Jeschke U., Knabl J., Heidegger H.H. (2020). Placental Galectin-2 Expression in Gestational Diabetes: A Systematic, Histological Analysis. Int. J. Mol. Sci..

[9] Juan J., Yang H. (2020). Prevalence, Prevention, and Lifestyle Intervention of Gestational Diabetes Mellitus in China. Int. J. Environ. Res. Public Health.

[10] Huang X., Liang W., Yang R., Jin L., Zhao K., Chen J., Shang X., Zhou Y., Wang X., Yu H. (2024). Variations in the *LINGO2* and *GLIS3* Genes and Gene-Environment Interactions Increase Gestational Diabetes Mellitus Risk in Chinese Women. Environ. Sci. Technol..

[11] Hedderson M.M. (2026). Gestational Diabetes Mellitus: Early Risk Factors, Prevention, Optimal Treatment, and Long-term Consequences for Mothers and Offspring: Reflections on 25 Years of Research: The 2025 Norbert Freinkel Award Lecture. Diabetes Care.

[12] Storozhuk M., Cherninskyi A., Maximyuk O., Isaev D., Krishtal O. (2021). Acid-Sensing Ion Channels: Focus on Physiological and Some Pathological Roles in the Brain. Curr. Neuropharmacol..

[13] Radu B.M., Dumitrescu D.I., Marin A., Banciu D.D., Iancu A.D., Selescu T., Radu M. (2014). Advanced type 1 diabetes is associated with ASIC alterations in mouse lower thoracic dorsal root ganglia neurons. Cell Biochem. Biophys..

[14] Kim S.-H., Liu M., Jin H.S., Park S. (2019). High Genetic Risk Scores of *ASIC2*, *MACROD2*, *CHRM3*, and *C2orf83* Genetic Variants Associated with Polycystic Ovary Syndrome Impair Insulin Sensitivity and Interact with Energy Intake in Korean Women. Gynecol. Obstet. Investig..

[15] Li L., Ding X., Wang X., Yao Q., Shao X., An X., Yan N., Jiang Y., Wang W., Shi L. (2018). Polymorphisms of IKZF3 Gene and Autoimmune Thyroid Diseases: Associated with Graves’ Disease but Not with Hashimoto’s Thyroiditis. Cell. Physiol. Biochem..

[16] Cai X., Qiao Y., Diao C., Xu X., Chen Y., Du S., Liu X., Liu N., Yu S., Chen D. (2014). Association between polymorphisms of the IKZF3 gene and systemic lupus erythematosus in a Chinese Han population. PLoS ONE.

[17] Fishilevich S., Nudel R., Rappaport N., Hadar R., Plaschkes I., Iny Stein T., Rosen N., Kohn A., Twik M., Safran M. (2017). GeneHancer: Genome-wide integration of enhancers and target genes in GeneCards. Database.

[18] Broz P., Pelegrín P., Shao F. (2019). The gasdermins, a protein family executing ceAll death and inflammation. Nat. Rev. Immunol..

[19] Zou J., Zheng Y., Huang Y., Tang D., Kang R., Chen R. (2021). The Versatile Gasdermin Family: Their Function and Roles in Diseases. Front. Immunol..

[20] Sarrio D., Colomo S., Moreno-Bueno G. (2024). Gasdermin-B (GSDMB) takes center stage in antibacterial defense, inflammatory diseases, and cancer. FEBS J..

[21] He H., Yi L., Zhang B., Yan B., Xiao M., Ren J., Zi D., Zhu L., Zhong Z., Zhao X. (2021). USP24-GSDMB complex promotes bladder cancer proliferation via activation of the STAT3 pathway. Int. J. Biol. Sci..

[22] Jiang H., Deng L., Lin Z., Yang K., Yang J., Zhao W., Gong W. (2024). GSDMB interacts with IGF2BP1 to suppress colorectal cancer progression by modulating DUSP6-ERK pathway. Int. Immunopharmacol..

[23] Saleh N.M., Raj S.M., Smyth D.J., Wallace C., Howson J.M., Bell L., Walker N.M., Stevens H.E., Todd J.A. (2011). Genetic association analyses of atopic illness and proinflammatory cytokine genes with type 1 diabetes. Diabetes Metab. Res. Rev..

[24] Kaur S., Mirza A.H., Overgaard A.J., Pociot F., Storling J. (2021). A Dual Systems Genetics Approach Identifies Common Genes, Networks, and Pathways for Type 1 and 2 Diabetes in Human Islets. Front. Genet..

[25] Pan S.-C., Huang C.-C., Chen B.-Y., Chin W.-S., Guo Y.L. (2023). Risk of type 2 diabetes after diagnosed gestational diabetes is enhanced by exposure to PM2.5. Int. J. Epidemiol..

[26] Li Y., Wang J., Yang S., Zhang S. (2021). Occurrence, health risks and soil-air exchange of phthalate acid esters: A case study in plastic film greenhouses of Chongqing, China. Chemosphere.

[27] Wang Z., Ma J., Wang T., Qin C., Hu X., Mosa A., Ling W. (2023). Environmental health risks induced by interaction between phthalic acid esters (PAEs) and biological macromolecules: A review. Chemosphere.

[28] Chen M., Niu Z., Zhang X., Zhang Y. (2024). Pollution characteristics and health risk of sixty-five organics in one drinking water system: PAEs should be prioritized for control. Chemosphere.

[29] Wang B., Wang H., Zhou W., Chen Y., Zhou Y., Jiang Q. (2015). Urinary excretion of phthalate metabolites in school children of China: Implication for cumulative risk assessment of phthalate exposure. Environ. Sci. Technol..

[30] Paoli D., Pallotti F., Dima A.P., Albani E., Alviggi C., Causio F., Dioguardi C.C., Conforti A., Ciriminna R., Fabozzi G. (2020). Phthalates and Bisphenol A: Presence in Blood Serum and Follicular Fluid of Italian Women Undergoing Assisted Reproduction Techniques. Toxics.

[31] Wang H., Chen R., Gao Y., Qu J., Zhang Y., Jin H., Zhao M., Bai X. (2023). Serum concentrations of phthalate metabolites in pregnant women and their association with gestational diabetes mellitus and blood glucose levels. Sci. Total Environ..

[32] James-Todd T., Ponzano M., Bellavia A., Williams P.L., Cantonwine D.E., Calafat A.M., Hauser R., Quinn M.R., Seely E.W., McElrath T.F. (2022). Urinary phthalate and DINCH metabolite concentrations and gradations of maternal glucose intolerance. Environ. Int..

[33] Martínez-Ibarra A., Martínez-Razo L.D., Vázquez-Martínez E.R., Martínez-Cruz N., Flores-Ramírez R., García-Gómez E., López-López M., Ortega-González C., Camacho-Arroyo I., Cerbón M. (2019). Unhealthy Levels of Phthalates and Bisphenol A in Mexican Pregnant Women with Gestational Diabetes and Its Association to Altered Expression of miRNAs Involved with Metabolic Disease. Int. J. Mol. Sci..

[34] Zhang Y., Zhao K., Jin L., Zhou Y., Shang X., Wang X., Yu H. (2024). MTNR1B gene variations and high pre-pregnancy BMI increase gestational diabetes mellitus risk in Chinese women. Gene.

[35] Lizárraga D., Gómez-Gil B., García-Gasca T., Ávalos-Soriano A., Casarini L., Salazar-Oroz A., García-Gasca A. (2024). Gestational diabetes mellitus: Genetic factors, epigenetic alterations, and microbial composition. Acta Diabetol..

[36] Zhang Y., Yang R., Wang X., Yu H. (2026). CDKN2A/2B gene variants and gene-environment interactions increase the risk of gestational diabetes mellitus in Chinese women. Front. Cell Dev. Biol..

[37] Wu Y., Zhang Y., Liu X., Liu J., He Z., Wei Y., Zeng Q., Guo R. (2025). Association between *KCNQ1* gene polymorphisms and gestational diabetes mellitus susceptibility in a Chinese population. Front. Endocrinol..

[38] Suthon S., Angkatavanich W., Mohamud S.H., Innang S., Songlilitchuwong S., Teerawattanapong N., Narkdontri T., Boriboonhirunsarn D., Tangjittipokin W. (2026). Genetic determinants of gestational diabetes mellitus in thai pregnant women: Role of *GCKR*, *CDKAL1*, *TCF7L2*, *NEDD1*, and *CMIP* variants. Ann. Med..

[39] Hryniewicka J., Buczyńska-Backiel A., Zbucka-Krętowska M., Krętowski A.J., Szelachowska M. (2026). Molecular Genetics of β-Cell Compensation in Gestational Diabetes Mellitus: Insights from CDKAL1, SLC30A8 and HHEX. Int. J. Mol. Sci..

[40] Zheng Y., Bian J., Hart J., Laden F., Soo-Tung Wen T., Zhao J., Qin H., Hu H. (2022). PM_2.5_ Constituents and Onset of Gestational Diabetes Mellitus: Identifying Susceptible Exposure Windows. Atmos. Environ..

[41] Sokkou S., Tzintros S.T., Konstantinidis I., Keramas A., Georgaki M.N., Stamoula E., Matsas A. (2025). Assessment of Environmental Risk Factors for Gestational Diabetes Mellitus: A Ten-Year Systematic Review and Meta-Analysis. J. Clin. Med..

[42] Ye B., Zhong C., Li Q., Xu S., Zhang Y., Zhang X., Chen X., Huang L., Wang H., Zhang Z. (2020). The Associations of Ambient Fine Particulate Matter Exposure During Pregnancy With Blood Glucose Levels and Gestational Diabetes Mellitus Risk: A Prospective Cohort Study in Wuhan, China. Am. J. Epidemiol..

[43] Diamanti-Kandarakis E., Bourguignon J.P., Giudice L.C., Hauser R., Prins G.S., Soto A.M., Zoeller R.T., Gore A.C. (2009). Endocrine-disrupting chemicals: An Endocrine Society scientific statement. Endocr. Rev..

[44] Sifakis S., Androutsopoulos V.P., Tsatsakis A.M., Spandidos D.A. (2017). Human exposure to endocrine disrupting chemicals: Effects on the male and female reproductive systems. Environ. Toxicol. Pharmacol..

[45] Schug T.T., Johnson A.F., Birnbaum L.S., Colborn T., Guillette L.J., Crews D.P., Collins T., Soto A.M., Vom Saal F.S., McLachlan J.A. (2016). Minireview: Endocrine Disruptors: Past Lessons and Future Directions. Mol. Endocrinol..

[46] Shoshtari-Yeganeh B., Zarean M., Mansourian M., Riahi R., Poursafa P., Teiri H., Rafiei N., Dehdashti B., Kelishadi R. (2019). Systematic review and meta-analysis on the association between phthalates exposure and insulin resistance. Environ. Sci. Pollut. Res. Int..

