# Supplementary material for: Associations of ASIC2, IKZF3, and GSDMB Variants with Gestational Diabetes Mellitus and Their Interactions with Environmental Exposures Among Chinese Women
Source: Biomolecules. 2026 Sep 21;16(9):1373. doi: 10.3390/biom16091373 (PMC13604340; doi:10.3390/biom16091373)
Supplement: Supplementary file 1 [file biomolecules-16-01373-s001.zip › biomolecules-4553901-supplementary.pdf]

Supplementary Methods: Detailed Statistical Analysis

Hardy–Weinberg equilibrium (HWE) was assessed for each SNP in the control group using a chi-square goodness-of-fit test implemented in an online HWE calculator for biallelic markers. Observed genotype counts were compared with those expected under HWE. For 1 degree of freedom, χ^2 values below the critical value of 3.84 were considered to indicate no significant deviation from HWE ($P > 0.05$). Haplotype frequencies for the three SNPs were estimated using the SHEsis program. The lowest frequency threshold (LFT) was left at the default setting of 0.03; therefore, haplotypes with estimated frequencies below 3% were not included in the haplotype distribution and association analyses.

GDM status was coded as 1 for GDM and 0 for controls. For interaction analyses, each SNP was coded according to the number of alternative (ALT) alleles (0, 1, or 2). The predefined $PM_{2.5}$ and O_3 exposure tertiles were treated as ordinal variables and coded as 1, 2, and 3 for low, medium, and high exposure, respectively. These variables were not analyzed as continuous pollutant concentrations or represented by separate dummy variables. Accordingly, ORs for interaction terms involving $PM_{2.5}$ or O_3 correspond to a one-level increase in the ordinal exposure variable (i.e., from low to medium or from medium to high exposure), rather than to a 1 μg/m^3 increase in the original pollutant concentration. Creatinine-normalized MIBP concentration (μg/g creatinine) was included as a continuous variable without further categorization or transformation. For regression-based analyses involving the combined T-T-T genetic pattern, participants carrying at least one T allele at each of rs12450465, rs2060941, and rs2305479 were coded as 1, whereas all other genotype combinations were coded as 0. No centering or standardization was performed for the genetic or environmental variables before construction of the interaction terms.

No single genetic model was prespecified as the primary model; therefore, the five genetic models were jointly considered in the multiple-testing correction for the single-gene association analyses. The Benjamini–Hochberg FDR procedure was applied separately within predefined analytical families, including single-gene association analyses, gene–gene interaction analyses, gene–air pollutant interaction analyses, haplotype–air pollutant interaction analyses, gene–MIBP-related interaction analyses, and haplotype–MIBP-related interaction analyses. Both nominal and FDR-adjusted *P*-values were reported for the unadjusted primary analyses.

In addition to the unadjusted models, multivariable-adjusted logistic regression models were fitted to evaluate the robustness of the observed associations to potential confounding. Maternal age, pre-pregnancy BMI, and recruitment region were included as covariates, with

recruitment region modeled as a categorical variable corresponding to Zunyi, Wuhan, and Beijing, with Zunyi used as the reference category for model parameterization. In the multivariable-adjusted higher-order interaction models, the corresponding lower-order terms were retained. These multivariable-adjusted models were considered sensitivity analyses. Because the Benjamini–Hochberg FDR correction was prespecified for the original unadjusted hypothesis families, the FDR-adjusted P-values reported for the primary analyses were derived from the unadjusted models, whereas adjusted P-values are reported for the multivariable-adjusted sensitivity analyses.

Supplementary Tables

Table S1. Literature-based evidence supporting the selection of candidate genes

Gene	Representative study	Key evidence	Rationale for inclusion in the present study
ASIC2	Kim SH, Liu M, Jin HS, Park S. Gynecol Obstet Invest. 2019;84(3):225–236. DOI: 10.1159/000493131	Genetic variation involving *ASIC2* was associated with insulin-resistance-related phenotypes, including higher fasting insulin and HOMA-IR, in Korean women.	*ASIC2* was considered a biologically plausible candidate because impaired insulin sensitivity and insulin resistance are important metabolic features of GDM.
IKZF3	Inshaw JRJ, Cutler AJ, Crouch DJM, Wicker LS, Todd JA. Diabetes Care. 2020;43(1):169–177. DOI: 10.2337/dc19-0803	*IKZF3* lies within a non-HLA region implicated in type 1 diabetes susceptibility and has potential roles in immune-cell development and function.	*IKZF3* was prioritized because of its location within a diabetes susceptibility region and its plausible immune-regulatory role.
GSDMB	Ram R, Mehta M, Nguyen QT, et al. J Immunol. 2016;196(7):3043–3053. DOI: 10.4049/jimmunol.1502056	*GSDMB* was identified within a type 1 diabetes susceptibility locus, and coding variants in this gene were considered potentially functional.	*GSDMB* was prioritized because of its diabetes-related genetic evidence and potential functional relevance.

Table S2. Sensitivity analyses of gene–gene, gene–air pollutant, and haplotype–air pollutant interactions with GDM in the overall study population (N = 1,564).

Model	Nominal *P*	Unadjusted OR(95%CI）	FDR-adjusted *P* [a]	Adjusted *P*	Adjusted OR(95% CI)
rs12450465 × rs2060941	0.01	1.304(1.066-1.594)	0.032	0.044	1.621（1.013-2.594）
rs12450465 × rs2305479	0.016	1.139(1.025-1.267)	0.032	0.296	1.143（0.890-1.468）
rs2060941 × rs2305479	0.17	1.156(0.940-1.422)	0.170	0.383	0.778（0.443-1.367）
rs12450465 × rs2060941 × rs2305479	0.037	1.168(1.010-1.350)	0.0493	0.301	1.148（0.884-1.491）
rs12450465 × $PM_{2.5}$	0.036	1.061(1.004-1.121)	0.054	0.257	0.899（0.747-1.081）
rs2060941 × $PM_{2.5}$	0.103	1.112(0.979-1.263)	0.115	0.581	0.908（0.646-1.278）
rs2305479 × $PM_{2.5}$	0.115	1.060(0.996-1.141)	0.115	0.355	0.907（0.738-1.115）
rs12450465 × O_3	2.0203×10^{-7}	1.157(1.095-1.222)	1.82×10^{-6}	0.130	0.865（0.717-1.044）
rs2060941 × O_3	0.016	1.17(1.030-1.328)	0.0288	0.415	0.866（0.613-1.224）
rs2305479 × O_3	1.82×10^{-4}	1.146(1.067-1.231)	0.000819	0.979	1.003（0.812-1.238）
rs12450465 × $PM_{2.5}$ × O_3	0.001	1.035(1.014-1.057)	0.003	0.366	1.116（0.879-1.418）
rs2060941 × $PM_{2.5}$ × O_3	0.07	1.051(0.996-1.110)	0.090	0.364	1.221（0.794-1.878）
rs2305479 × $PM_{2.5}$ × O_3	0.012	1.039(1.008-1.071)	0.027	0.559	1.084（0.827-1.421）
T-T-T × $PM_{2.5}$	0.937	1.004(0.908-1.111)	0.937	0.514	1.138（0.772-1.677）
T-T-T × O_3	0.000014	1.254(1.132-1.390)	0.000042	0.485	1.150（0.777-1.702）
T-T-T × $PM_{2.5}$ × O_3	0.019	1.044(1.007-1.082)	0.0285	0.188	0.724（0.447-1.171）

[a] FDR-adjusted *P* values were derived from the nominal *P* values of the unadjusted analyses using the Benjamini–Hochberg procedure.

Table S3. Sensitivity analyses of MIBP–related associations and interaction terms with GDM in the MIBP subset (N = 289).

Model	Nominal *P*	Unadjusted OR (95%CI）	FDR-adjusted *P* [a]	Adjusted *P*	Adjusted OR (95% CI)
MIBP	0.034	1.002(1.000-1.004)	0.0884	0.018	1.003 (1.000-1.005)
rs12450465 × MIBP	0.031	1.002(1.000-1.003)	0.0884	0.836	0.999 (0.994-1.005)
rs2060941 × MIBP	0.142	1.011(0.996-1.027)	0.3077	0.928	1.001 (0.982-1.020)
rs2305479 × MIBP	0.683	0.999(0.992-1.005)	0.7399	0.448	0.997 (0.989-1.005)
rs12450465 × MIBP × $PM_{2.5}$	0.008	1.001(1.000-1.002)	0.0520	0.105	1.002 (1.000-1.005)
rs2060941 × MIBP × $PM_{2.5}$	0.291	1.003(0.997-1.009)	0.5395	0.821	0.998 (0.976-1.019)
rs2305479 × MIBP × $PM_{2.5}$	0.592	0.999(0.996-1.002)	0.7399	0.701	1.002 (0.992-1.013)
rs12450465 × MIBP × O_3	0.012	1.001(1.000-1.002)	0.0520	0.945	1.000 (0.992-1.007)
rs2060941 × MIBP × O_3	0.332	1.003(0.997-1.010)	0.5395	0.158	0.974 (0.939-1.010)
rs2305479 × MIBP × O_3	0.991	1.000(0.996-1.004)	0.9910	0.903	1.001 (0.999-1.013)
rs12450465 × MIBP × $PM_{2.5}$ × O_3	0.008	1.001(1.000-1.001)	0.0520	0.484	1.003 (0.994-1.012)
rs2060941 × MIBP × $PM_{2.5}$ × O_3	0.486	1.001(0.998-1.003)	0.7020	0.473	1.029 (0.951-1.114)
rs2305479 × MIBP × $PM_{2.5}$ × O_3	0.648	1.000(0.998-1.002)	0.7399	0.429	0.991 (0.969-1.013)
T-T-T × MIBP	0.045	1.002(1.000-1.004)	0.0450	0.903	1.001 (0.982-1.021)
T-T-T × MIBP × $PM_{2.5}$	0.021	1.001(1.000-1.003)	0.0293	0.871	1.002 (0.979-1.026)
T-T-T × MIBP × O_3	0.022	1.001(1.000-1.003)	0.0293	0.318	1.017 (0.984-1.052)
T-T-T × MIBP × $PM_{2.5}$ × O_3	0.015	1.001(1.000-1.001)	0.0293	0.097	1.010 (0.998-1.023）

[a] FDR-adjusted *P* values were derived from the nominal *P* values of the unadjusted analyses using the Benjamini–Hochberg procedure.

Table S4. The influence of physical characteristics on the risk of developing GDM

**Factor**		**GDM (N=701)**	**Control (N=863)**	***P*-value**	**OR(95% CI)**
Age	≤28	202	278	0.147	0.852(0.686-1.058)
	＞28	499	585		
Height	≤158	223	228	0.149	1.177(0.943-1.467)
	＞158	478	575		
Gestational age	≤24	5	5	0.741	1.233(0.355-4.275)
	＞24	696	858		
Pre-pregnancy BMI	≤28	46	23	0.00019	2.565(1.539-4.275)
	＞28	655	840		

Table S5. Hardy–Weinberg equilibrium analysis of the three SNPs in the control group

SNP	Genotype	Observed genotype count	Expected genotype count	Minor allele	MAF	χ^2	HWE
	CC	122	130.82				
rs12450465	CT	428	410.36	C	0.389	1.59	Consistent
	TT	313	321.82				
	GG	769	766.84				
rs2060941	GT	89	93.32	T	0.057	1.85	Consistent
	TT	5	2.84				
	CC	491	491.08				
rs2305479	CT	320	319.84	T	0.246	0	Consistent
	TT	52	52.08				

MAF, minor allele frequency; HWE, Hardy–Weinberg equilibrium. MAF was calculated from genotype counts in the control group. HWE was assessed using a chi-square goodness-of-fit test. For 1 degree of freedom, a χ^2 value < 3.84 corresponds to $P > 0.05$; therefore, SNPs with $\chi^2 < 3.84$ were considered consistent with HWE.

Table S6. Genotype and allele frequencies by recruitment city and GDM status

Table S6a. Genotype frequencies by recruitment city and GDM status

SNP	Group	Zunyi n (%) (Control n=364; GDM n=323)	Wuhan n (%) (Control n=406; GDM n=235)	Beijing n (%) (Control n=93; GDM n=143)
rs12450465				
CC	Control	50 (13.7)	55 (13.5)	17 (18.3)
CT	Control	169 (46.4)	211 (52.0)	48 (51.6)
TT	Control	145 (39.8)	140 (34.5)	28 (30.1)
CC	GDM	41 (12.7)	34 (14.5)	15 (10.5)
CT	GDM	145 (44.9)	108 (46.0)	65 (45.5)
TT	GDM	137 (42.4)	93 (39.6)	63 (44.1)
rs2060941				
GG	Control	323 (88.7)	360 (88.7)	86 (92.5)
GT	Control	38 (10.4)	44 (10.8)	7 (7.5)
TT	Control	3 (0.8)	2 (0.5)	0 (0.0)
GG	GDM	278 (86.1)	205 (87.2)	118 (82.5)
GT	GDM	44 (13.6)	28 (11.9)	24 (16.8)
TT	GDM	1 (0.3)	2 (0.9)	1 (0.7)
rs2305479				
CC	Control	207 (56.9)	232 (57.1)	52 (55.9)
CT	Control	135 (37.1)	151 (37.2)	34 (36.6)
TT	Control	22 (6.0)	23 (5.7)	7 (7.5)
CC	GDM	178 (55.1)	126 (53.6)	59 (41.3)
CT	GDM	129 (39.9)	95 (40.4)	70 (49.0)
TT	GDM	16 (5.0)	14 (6.0)	14 (9.8)

Table S6b. Allele frequencies by recruitment city and GDM status

SNP	Allele	Group	Zunyi n (%)	Wuhan n (%)	Beijing n (%)
rs12450465	C	Control	269 (37.0)	321 (39.5)	82 (44.1)
	T	Control	459 (63.0)	491 (60.5)	104 (55.9)
	C	GDM	227 (35.1)	176 (37.4)	95 (33.2)
	T	GDM	419 (64.9)	294 (62.6)	191 (66.8)
rs2060941	G	Control	684 (94.0)	764 (94.1)	179 (96.2)
	T	Control	44 (6.0)	48 (5.9)	7 (3.8)
	G	GDM	600 (92.9)	438 (93.2)	260 (90.9)
	T	GDM	46 (7.1)	32 (6.8)	26 (9.1)
rs2305479	C	Control	549 (75.4)	615 (75.7)	138 (74.2)
	T	Control	179 (24.6)	197 (24.3)	48 (25.8)
	C	GDM	485 (75.1)	347 (73.8)	188 (65.7)
	T	GDM	161 (24.9)	123 (26.2)	98 (34.3)

Table S7. Association of Different Genetic Models of rs12450465, rs2060941, and rs2305479 with the GDM Risk.

SNP	GENE	REF/ALT	Model	Unadjusted OR(95%CI)	*P* value	FDR-adjusted *P* [a]
rs12450465	*ASIC2*	C/T	dominant (TT+CT vs.CC)	1.118 (0.834-1.498)	0.456	0.684
			recessive (TT vs.CT+CC)	1.262 (1.029-1.548)	0.026	0.124
			allelic (T vs.C)	1.157 (1.000-1.339)	0.05	0.124
			heterozygous (CT vs.CC)	1.007 (0.740-1.371)	0.964	0.982
			homozygous (TT vs.CC)	1.269 (0.926-1.740)	0.139	0.232
rs2060941	*IKZF3*	G/T	dominant (TT+GT vs.GG)	1.361 (1.007-1.839)	0.044	0.124
			recessive (TT vs.GT+GG)	0.985 (0.263-3.681)	0.982	0.982
			allelic (T vs.G)	1.317 (0.991-1.750)	0.058	0.124
			heterozygous (GT vs.GG)	1.380 (1.015-1.877)	0.039	0.124
			homozygous (TT vs.GG)	1.024 (0.274-3.828)	0.972	0.982
rs2305479	*GSDMB*	C/T	dominant (TT+CT vs.CC)	1.229 (1.006-1.501)	0.043	0.124
			recessive (TT vs.CT+CC)	1.044 (0.690-1.581)	0.837	0.982
			allelic (T vs.C)	1.150 (0.979-1.351)	0.088	0.165
			heterozygous (CT vs.CC)	1.243 (1.009-1.531)	0.041	0.124
			homozygous (TT vs.CC)	1.145 (0.749-1.749)	0.532	0.725

[a] FDR-adjusted *P* values were derived from the nominal *P* values of the unadjusted analyses using the Benjamini–Hochberg procedure.

Table S8. Results on the impact of gene-environment interactions on the risk of developing GDM

Model	Unadjusted OR(95%CI)	Nominal *P*	FDR-adjusted *P* [a]
rs12450465 × $PM_{2.5}$	1.061(1.004-1.121)	0.036	0.054
rs2060941 × $PM_{2.5}$	1.112(0.979-1.263)	0.103	0.115
rs2305479 × $PM_{2.5}$	1.060(0.996-1.141)	0.115	0.115
rs12450465 × O_3	1.157(1.095-1.222)	2.0203×10^{-7}	1.82×10^{-6}
rs2060941 × O_3	1.17(1.030-1.328)	0.016	0.0288
rs2305479 × O_3	1.146(1.067-1.231)	1.82×10^{-4}	0.000819
rs12450465 × $PM_{2.5}$ × O_3	1.035(1.014-1.057)	0.001	0.003
rs2060941 × $PM_{2.5}$ × O_3	1.051(0.996-1.110)	0.07	0.090
rs2305479 × $PM_{2.5}$ × O_3	1.039(1.008-1.071)	0.012	0.027

[a] FDR-adjusted *P* values were obtained by applying the Benjamini–Hochberg procedure to the nominal *P* values from the unadjusted primary gene–air pollutant interaction analyses.

Table S9. Association between haplotypes （rs12450465- rs2060941- rs2305479）and GDM incidence

Haplotype	Unadjusted OR(95%CI)	Nominal *P*	FDR-adjusted *P* [a]
C-G-C	0.856(0.730-1.003)	0.054135	0.13534
C-G-T	0.997(0.758-1.311)	0.982682	0.98268
T-G-C	0.990(0.858-1.142)	0.893	0.98268
T-G-T	1.122(0.906-1.389)	0.291959	0.48660
T-T-T	1.741(1.208-2.509)	0.00266	0.01330

[a] FDR-adjusted *P* values were derived from the nominal *P* values of the unadjusted analyses using the Benjamini–Hochberg procedure.

Supplementary Figures

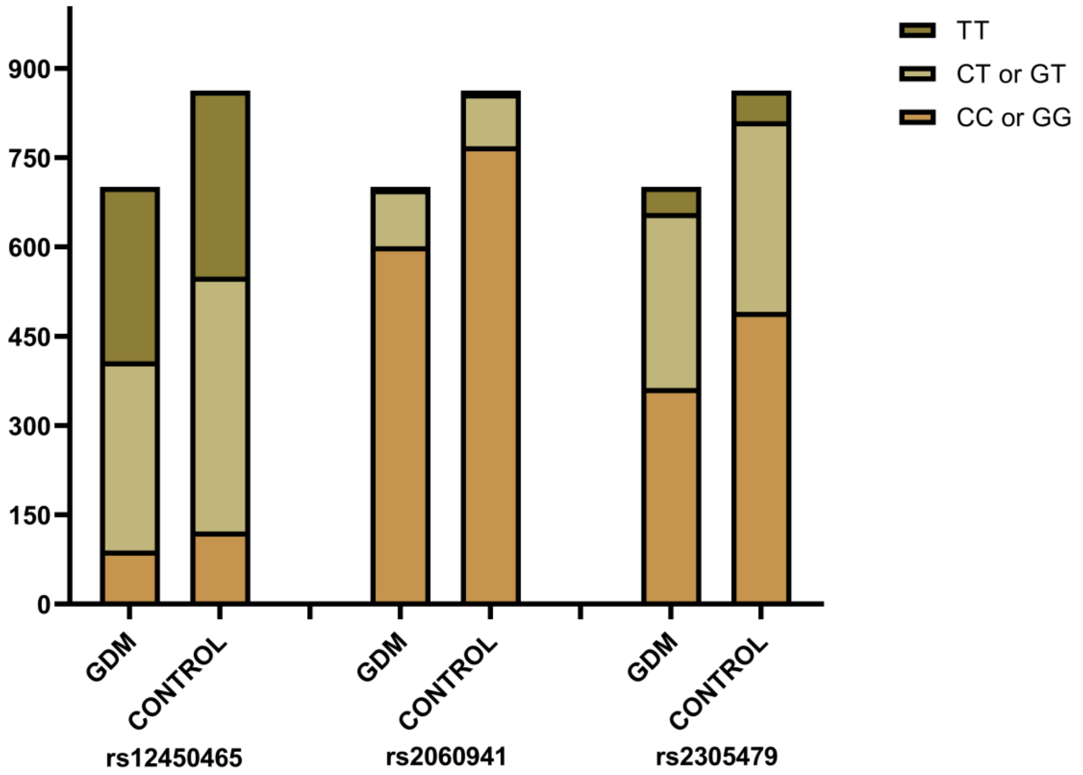

Figure S1. Genotype Distribution of rs12450465, rs2060941, and rs2305479 Between the GDM and Control Groups. Comparison of the genotype distributions of rs12450465, rs2060941, and rs2305479 between the GDM and control groups. The figure shows the frequency distributions of the different genotypes at each genetic locus in the two groups.

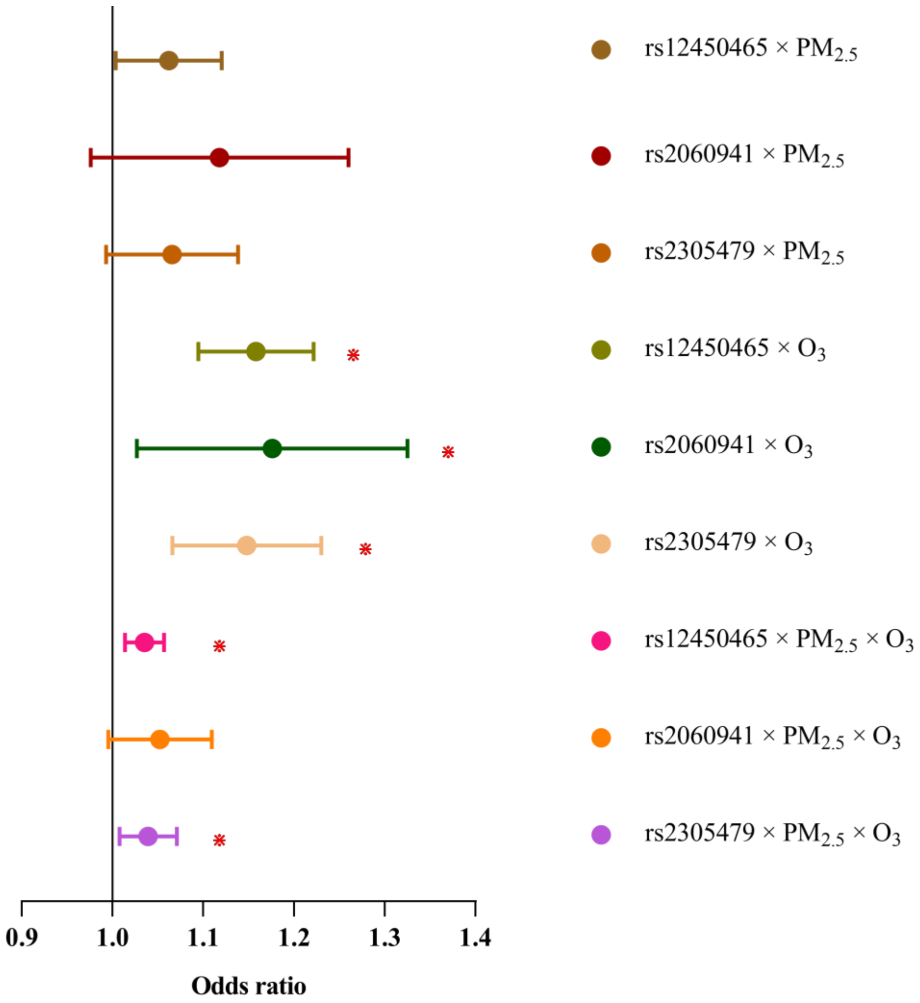

Figure S2. Associations of gene–environment interaction terms with GDM. Odds ratios and 95% confidence intervals were estimated from the unadjusted primary analyses for interaction terms involving rs12450465, rs2060941, or rs2305479 and $PM_{2.5}$ and/or O_3. Asterisks indicate interaction terms that remained statistically significant after Benjamini–Hochberg FDR correction (FDR-adjusted $P < 0.05$).

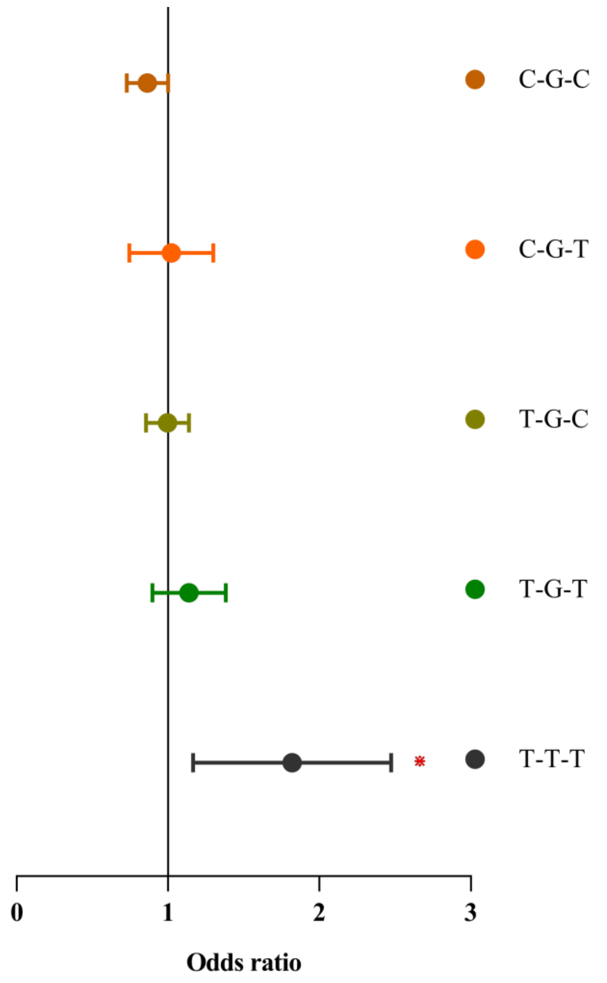

Figure S3. Associations of rs12450465-rs2060941-rs2305479 haplotypes with GDM. Odds ratios and 95% confidence intervals were estimated from the unadjusted primary haplotype association analyses. Asterisks indicate haplotype associations that remained statistically significant after Benjamini–Hochberg FDR correction (FDR-adjusted $P < 0.05$).
